# Mobility indexes of Cu, Pb, and Zn in soil ecosystems with various levels of metal contamination (in Poland)

**DOI:** 10.1007/s10661-023-11069-0

**Published:** 2023-03-23

**Authors:** Krystyna Niesiobędzka

**Affiliations:** Faculty of Building Installations, Hydrotechnics and Environmental Engineering, Warsaw University of Technology, ul. Nowowiejska 20, 00-653 Warsaw, Poland

**Keywords:** Soil ecosystem, Pollution, Cu, Pb, Zn, Mobility indexes

## Abstract

The aim of the study was to investigate and compare the soils of three groups of grassland ecosystems with varying degrees of heavy metal (Cu, Pb, and Zn) pollution as well as estimating of mobility on the basis of calculated mobility indexes (MI) expressed as a percentage of the EDTA-extracted forms of metals in their total content. A total of 55 surface soil samples were collected from various areas of Poland: urban soils, rural soils, and soils along communication routes. Heavy metal concentrations were determined in solutions after wet mineralization (using a mixture of acids) by atomic absorption spectrometry (AAS) with flame atomization. To isolate the mobile forms of metals in soils, a one-step extraction method was used with 0.05-M EDTA solution. The ranges of Cu, Pb, and Zn concentrations in soils were varied, respectively: 6.7–47.6, 61.1–563.9, and 86.4–644.5 mg·kg^−1^ (A); 5.7–39.8, 13.56–45.71, and 16.3–119.6 mg·kg^−1^ (B); and 1.0–195.8, 19.2–310.2, and 27.4–894.1 mg·kg^−1^ (C). The average values of mobility indexes of Cu, Pb, and Zn were at the level of 40.9, 33.5, and 22.2% (A); 23.2, 27.1, and 25.9% (B); and 37.5, 34.3, and 30.7% (C). Studies have shown that metals derived from anthropogenic sources are characterized by greater mobility compared to metals of natural origin (lithological associated with the ground). The inclusion of metal mobility indices in the environmental monitoring strategy may minimize errors in assessing the actual risk associated with the potential uptake of these metals by plants and incorporation into circulation.

## Introduction

Heavy metals in the environment rarely occur in free form or as individual ions, while the typical forms in which they are most often accumulated in soils are soluble, ion exchange, and non-exchangeable forms (permanently associated with soil components). Speciation analysis is used to reliably assess the impact of metals on the soil environment due to the fact that their forms have different release capacities into solution and thus different toxicity. Determining the type and percentage of these forms in the total metal content brings extremely important information for assessing the bioavailability of metals for living organisms, as well as the possibilities of their transport in the environment. The assessment of the state of the environment and long-term ecological threats based on the total metal content has usually led to an overestimation of the degree of risk. Hence, research aimed at developing analytical methods that allow estimating the share of mobile, ecologically dangerous, and easily migrating forms in the trophic chain has become a necessity. Bioavailability is a term often used to describe the ability to release a metal in a form in which it is readily available to living organisms and easily migrates into the trophic chain. Mobility is inextricably linked to the concept of bioavailability which is determined by the mobility index (MI) expressed as a percentage of the bioavailable forms of the metal in the total content of this metal in the soil.

There has been a keen interest in metallic pollution in various environmental components for many years. An important and rich group are articles devoted to speciation studies of metals and issues of selection of the best extraction reagent to assess the bioavailability of metals in the soil environment, because there is no universal method enabling a uniform way of determining biologically available fractions and their quantitative assessment. Usually, those metal forms whose concentrations correlate most strongly with their concentrations in plants are seen as bioavailable and determine the most useful extraction technique (Gupta & Sinha, 2007; Ure, 1996; Ure et al., 1993). Guided by the above premises and other reports from the world of science (Duplay et al., 2014; Rao et al., 2008, Korzeniowska & Stanisławska-Golubiak, 2013, McBride et al., 2004; Pueyo et al., 2004; Hammer & Keller, 2002; Lock & Janssen, 2003), one-stage extraction with the use of 0.05-M EDTA solution as the extractant was used to study bioavailable forms of metals. The author’s previous publications (Niesiobedzka, 2012, 2016, 2018) refer to the problems of heavy metal speciation in the soil environment, the influence of soil parameters on the forms of metal occurrence, and their bioavailability. Guided by the above premises and other reports from the world of science (Duplay et al., 2014; Rao et al., 2008; Korzeniowska & Stanislawska-Golubiak, 2013; McBride et al., 2004; Pueyo et al., 2004), Vázquez et al. (2016) in the study were focused on the assessment of single and sequential extraction methods to predict the bioavailability of metals in the vineyard soil-grapevine system. The modified BCR sequential extraction method and two single-step extraction methods based on the use of EDTA and acetic acid were applied to differently amended vineyard soils. Anju and Banerjee (2011) used a single extraction with EDTA as well as a sequential extraction scheme (BCR method) to assess the potential mobility of Cd, Pb, and Zn in soil (number of samples *n* = 23). The amount of Cd, Pb, and Zn extracted by EDTA and their total concentrations showed linear positive correlation. A different single extraction procedures (CH3COOH, Na2EDTA, CaCl2, NH4NO3, deionized water) and pseudo-total digestion (aqua regia) were applied to determine major (Al, Fe, K, Mn, Na, P, S, and Si) and trace (Cd, Co, Cr, Cu, Mo, Ni, Pb, V, and Zn) element bioavailability in a topsoil from the experimental vineyard (“Radmilovac,” Belgrade, Serbia) (Milićević et al., 2017). It is necessary to determine potentially available metal fractions for plants using speciation analysis methods to forecast ecological effects. Therefore, it is justified to estimate the mobility of metals in the soil environment depending on the degree of soil contamination and the form of metals deposited in it, which are derived from the type of pollution source.

## Materials and methods

### Study area

**Fig. 1:**
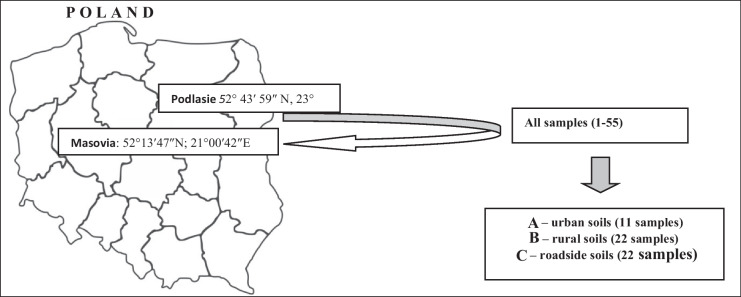
Sampling area (Poland—two provinces: Mazovia and Podlasie)

The research covered top soil layers (0–10 cm) representing three groups of grassland ecosystems differentiated by the location and impact of various sources of pollution: 11 samples of urban soils (called A ecosystem), 22 samples of rural soils (B ecosystem), and 22 samples of soils along communication routes (C ecosystem). The choice so varied in terms of the degree of exposure to anthropopressure was to investigate whether the nature of the metal source and the origin of these elements (natural or anthropogenic) in soil affect their mobility tested in the EDTA extraction test. Urban soil samples were taken from the Warsaw agglomeration (21°02′E, 52°11′N), affected by various sources of pollution (emission of industrial and automotive) typical for large cities (Fig. 1). The samples of rural soil samples were collected from the area of three poviats in northern Mazovia: Ciechanow, Pultusk, and Plonsk. These were areas after agricultural land, no longer covered by agrotechnical measures and excluded from farming (wasteland), and agricultural land left without human intervention (fallow). The soil samples of C ecosystem were taken (at a distance of 5–10 m from the road edge) along three communication roads of varying intensity: national road E77 (20°54′E, 52°21′N**)** and two provincial road—DW618 (21°06′E, 52°43′N) and DW689 (23°35′E, *5*2°44′N).

### Sampling and analysis methods

To isolate the forms of bioavailable metals in soils, a one-step extraction method was used with 0.05-M EDTA solution in accordance with the procedure described by Ure (1996). To this end, 100 cm^3^ of EDTA solution was added to 10-g soil samples (air-dry) and shaken on a shaker for 1 h. The suspension was then passed through a filter and the filtrate subjected to spectrometric analysis for determination of metals (Cu, Pb, and Zn) in the solution after extraction. The metal concentrations in the filtrate were converted to the dry soil mass and reported as the concentrations of the forms extracted with EDTA (Ure, 1996).

### Apparatus

Copper, lead, and zinc concentrations were determined in solutions after mineralization by atomic absorption spectrometry with flame atomization. A 100-mm burner fed with a stoichiometric mixture of air and medical acetylene (acetylene A) was used. The flame temperature was 2100–2300 ℃. As a radiation source, hollow cathode lamps made by Philips were used. The spectrophotometer was controlled by Unicam Atomic Absorption—Data Station 1.7 by Unicam. Detection limits for copper, lead, and zinc per mg·kg^−1^ of soil dry matter were 0.03, 0.05, and 0.01, respectively. In order to check the correctness of the analysis and the measurement accuracy, the reference material TILL-3 (Certificate of Analysis, 1995) with known metal content (Cu, 22 mg·kg^−1^; Pb, 26 mg·kg^−1^; and Zn, 56 mg·kg^−1^) was analyzed. The recovery rate of Cu, Pb, and Zn was satisfactory. The error resulting from the comparison of the obtained values did not exceed 10% for three elements.

### Analytical methods

For all laboratory analyses, methods of analytical determinations and conversion techniques were presented by Ostrowska et al. (1991) and Dobrzanski et al. (1987). All analysis results were expressed on the dry weight of the sample. As part of the physicochemical analysis of soil samples, the following determinations were made:

- Active soil acidity (pH_H2O_): potentiometric method
- Organic matter content: by weight method (based on loss of dry mass of the sample during roasting at 550 ℃
- Basic exchange cations: determination of cations (Ca^2+^, Mg^2+^, K^+^, Na^+^) in 1-M ammonium acetate extract with pH = 6.8 by AES
- Hydrolytic acidity (H_h_): Kappen method
- Total concentration of heavy metals (Cu, Pb, and Zn): determination in solution after sample mineralization with a mixture of acids (HNO_3_ and HClO_4_) by the AAS method (using a PU 9100X/74 atomic absorption spectrometer with Philips flame atomization)

Based on the results of analytical determinations, the following were calculated: sum of basic exchangeable cations (S) and capacity of sorption complex (T) - as H_h_ + S. Pure chemical reagents for analysis were used to conduct the tests. Heavy metal standard solutions (Cu, Pb, and Zn) were prepared from Merck analytical ampoules. Double distilled water was used to prepare all solutions.

### Statistical analysis of results

For statistical analysis, the computer programs Excel/Windows 10 and Statistica 13.1 were used. The following statistical values were determined:

- Average, minimum, and maximum values of the analyzed parameters, as well as the median and standard deviation
- Pearson correlation coefficients between concentrations of metals extracted by EDTA and total concentrations of metals in soils for each of the three groups of ecosystems (A, B, C), which was treated as three independent sets

From a scientific point of view, it was necessary to the formulation of an answer to the following working questions:

1. Did the differences in levels of Cu, Pb, and Zn accumulation soils between three groups of ecosystems (A, B, and C) were statistically significant?
2. Did the concentrations of EDTA-extracted metals in soils show statistically significant differences between three groups of ecosystems (A, B, C)?
3. Did the metal mobility indexes differ significantly between three groups of ecosystems (A, B, C)?

To check whether the objects selected for testing show statistically significant differences in the matter of pollution and metal mobility between groups, ANOVA (analysis of variance) was used for a single classification examining the effect of a classifying (grouping) factor on the value of the measurable characteristic tested. To be able to indicate which group means differ significantly from the others, additional tests were carried out, comparing selected pairs of group means using the NIR (least significant difference (LSD)) method.

## Results and discussion

The results of the physicochemical analysis of soil samples are shown in Table 1. The pH values of analyzed soils varied and ranged from 6.98 to 7.82 (urban soils), 3.35 to 7.98 (rural soils), and 5.70 to 8.20 (roadside soils). Mostly, however, samples of urban and roadside soils were alkaline with a median of 7.64 and 7.60, respectively, and a standard deviation of 0.22 and 0.64.

**Table 1:** Physicochemical characteristic of soils

Soil parameters					
Values	pH	OM (%)	Kh (cmol·kg^−1^)	S (cmol·kg^−1^)	T (cmol·kg^−1^)
**A ecosystem**					
Average	7.61	7.32	1.19	2.32	3.52
Median	7.64	5.96	1.18	2.33	3.35
Min	6.98	2.54	0.83	1.33	2.23
Max	7.82	16.77	1.62	4.21	5.45
SD	0.22	3.93	0.25	0.92	0.96
**B ecosystem**					
Average	5.66	6.47	1.90	15.19	17.09
Median	5.54	6.33	1.55	14.20	16.16
Min	3.35	2.08	0.83	5.54	6.76
Max	7.98	16.40	3.79	26.50	27.33
SD	1.31	3.41	0.79	5.32	5.09
**C ecosystem**					
Average	7.37	7.28	8.69	26.82	35.51
Median	7.60	5.98	7.50	25.80	34.05
Min	5.70	0.52	3.75	3.00	7.50
Max	8.20	39.58	25.50	49.80	58.60
SD	0.68	7.94	5.84	16.67	15.47

### The total and EDTA-extracted form concentrations of metals in soils

The values of total concentrations of copper, lead, and zinc and their EDTA-extracted forms (bioavailable forms) are summarized in Table 2. The average value of copper concentration in urban soils (A) was 28.6 mg·kg^−1^ DM with a median of 39.6 mg·kg^−1^, in rural soils (B), the average copper concentration was almost twice lower with a correspondingly lower median value of 14.8 mg·kg^−1^, and in roadside soils (C), copper accumulation was the highest with an average concentration of 38.1 mg·kg^−1^ and median 14.7 mg·kg^−1^. In general soil samples collected along the E-77 communications, route E-77 showed a high accumulation of this metal, indicating the III-IV degree of pollution (Kabata-Pendias et al., 1993). In most cases, the urban soils showed the concentration of copper at the level corresponding to the I-II degree of pollution. Rural soil samples, with few exceptions, did not exceed the limit values of natural copper concentrations in soils (Kabata-Pendias et al., 1995). The ranges of lead concentration in the tested environmental samples were very diverse and amounted to 61.1–563.9 mg·kg^−1^ in urban soils, 13.6–45.7 mg·kg^−1^ in rural soils, and 19.2–310.2 mg·kg^−1^ in roadside soils with average values of 213.8, 34.8, and 88.6 mg·kg^−1^, respectively. In rural soil samples, lead concentrations did not exceed the limit values of natural lead concentrations typical for Polish soils. In the group of roadside soil samples, the highest concentration values were found for samples taken along the E-77 national road, indicating the II degree of lead pollution. In soil samples taken on the DW618 road section, lead concentrations did not exceed the limits of natural levels. The average concentration of zinc in urban soils was 390.2 mg·kg^−1^ with a median of 537.3 mg·kg^−1^. In rural soils, the average zinc concentration was more than ten times lower (42.3 mg·kg^−1^) with a median of 24.8 mg·kg^−1^. In roadside soils, zinc accumulation was estimated at 251.6 mg·kg^−1^ with a median of 122.1 mg·kg^−1^. The highest concentration values were recorded in soil samples representing A ecosystem, which significantly exceeded the limit values of natural concentrations of this element in soil, in most cases 5 and 6 times, indicating the II-III degree of pollution (Kabata-Pendias et al., 1993). In the case of roadside soils, the highest zinc concentration values (819.9 mg·kg^−1^) were recorded for soil samples taken along the E-77 national road with the highest traffic intensity. In soil samples collected on the DW618 road section, zinc concentrations were lower and ranged between 91.3 and 358.6 mg·kg^−1^ DM, indicating the II degree of pollution (Kabata-Pendias et al., 1993). In general, the mean values of total concentrations in the top layers of soils representing ecosystem C were much higher than in other soils along the expressways (Trasa Lazienkowska, Wislostrada, and the exit route from Warsaw), described in other publications (Niesiobedzka, 2007). Similar studies were conducted by Wang et al. (2014), Yan et al. (2012), and Zhang (2011), focusing their attention on the accumulation of metallic impurities in roadside soils. The highest values of EDTA-extracted form concentrations of metals, attesting to their high bioavailability, were found in urban (Cu and Pb) and roadside (Zn) soils. Xu et al. (2009) provide very wide ranges of concentrations of bioavailable forms of metals: 8.12–70.33 mg·kg^−1^ (Cu), 3.16–90.33 mg·kg^−1^ (Pb), and 10.24–106.85 mg·kg^−1^ (Zn), indicating a large diversity of soils in terms of the degree of contamination and physicochemical properties.

**Table 2:** The total and the EDTA-extractable form concentrations of heavy metals in soils

	Total concentration of metals. (mg·kg^−1^)			EDTA-extractable forms concentration of metals. (mg·kg^−1^)		
Values	Cu	Pb	Zn	Cu	Pb	Zn
**A ecosystem**						
Average	28.6	213.8	390.2	10.9	64.3	66.7
Median	39.6	257.9	537.3	15.4	83.8	100.0
Min	6.7	61.1	84.6	3.1	19.0	26.8
Max	47.6	563.9	644.5	18.6	127.2	99.9
SD	16.1	141.4	230.3	5.7	34.1	21.1
**B ecosystem**						
Average	15.8	34.8	42.3	3.8	8.7	12.8
Median	14.8	37.6	24.8	3.4	7.2	5.5
Min	5.7	13.6	16.5	1.1	4.7	2.5
Max	39.8	45.7	119.6	11.5	17.7	59.0
SD	7.6	8.8	32.7	2.5	4.1	14.8
**C ecosystem**						
Average	38.1	88.6	251.6	9.1	25.7	75.9
Median	14.7	59.6	122.1	7.9	19.1	43.2
Min	1.0	19.2	27.4	0.2	5.9	5.9
Max	195.8	310.2	894.1	36.1	59.1	263.9
SD	57.1	71.9	275.0	9.7	15.8	81.1

### Mobility indexes of metals

**Fig. 2:**
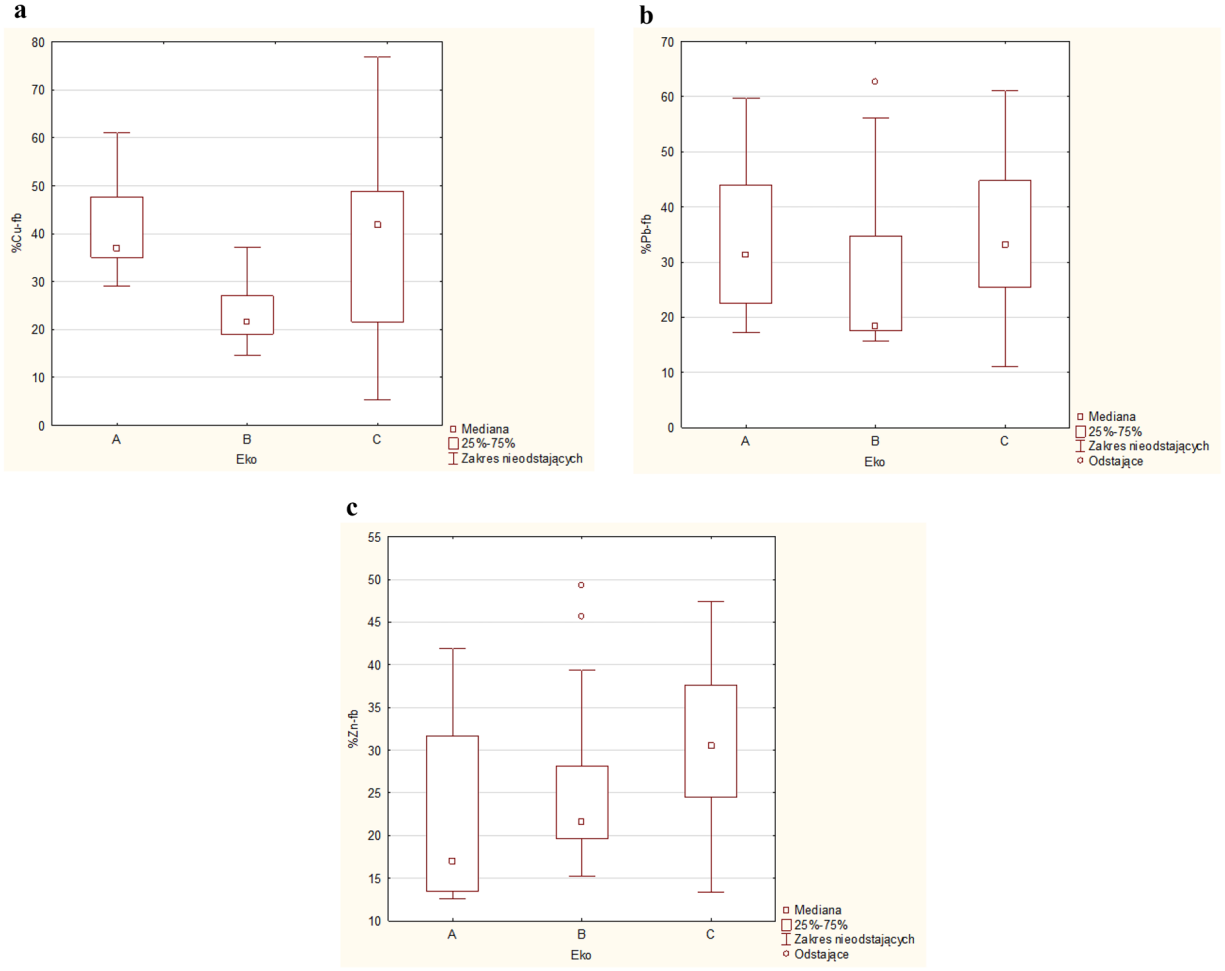
The percentage of EDTA-extractable forms (%Cu-fb, %Pb-fb, %Zn-fb) in soils (MI_Cu_, MI_Pb_, MI_Zn_)

The average values of copper mobility indexes in the three groups of ecosystems A, B, and C (Fig. 2) were 40.9%, 23.2%, and 37.5%, respectively, with the following ranges of variability: 29.0–61.0%, 7–37.1%, and 5.3–76.8%. The highest median value was recorded for roadside soil samples (C, 41.8%), while the lowest for rural soil samples (B, 21.5%), with the median percentage of copper bioavailable forms for urban soil samples (A) which was 37.0%.

The average values of Pb mobility indexes in urban soil samples were at the level of 33.5% (A ecosystem), 27.1% in rural soil samples (B), and 34.3% in roadside soil samples (C), and their ranges varied depending on the examined object: 17.2–59.8% (for A), 15.6–62.7% (for B), and 11.1–61.1% (for C). Soils from A ecosystem (31.4%) and C ecosystem (34.3%) were characterized by a very similar median value, while for soil samples of B ecosystem, the median value was at the level of 18.3%.

The average value of mobility index (MI, percentage of EDTA-extracted form of zinc in its total content in soil) for three groups of ecosystems (A, B, C) reached the following values, respectively: 22.2%, 25.0%, and 30.7%. The median value was 17.0, 21.6, and 30.5%. The ranges of variability were quite large and were (A, B, C) 12.6–41.9, 15.2–49.3, and 13.4–47.4%, respectively. The lowest median values of MI for copper and lead in rural soils indicate their lowest mobility and bioavailability. Zinc, which showed lower mobility in A ecosystem than in B ecosystem, is puzzling. It was probably influenced by other factors, such as soil pH and related hydrolytic acidity. The average pH value of soils of B ecosystem was almost 2 units lower than the pH of soils representing A ecosystem. Lower pH values and higher values of hydrolytic acidity of soils may be responsible for easier activation of zinc resulting in an increase in the share of its bioavailable forms under reduced pH conditions (Niesiobedzka, 2018).

The mobility index (MI) of the metals tested changed in the following order:

- MI_Cu_: A ecosystem (40.92%) > C ecosystem (37.53%) > B ecosystem (23.23%)
- MI_Pb_: C ecosystem (34.33%) > A ecosystem (33.52%) > B ecosystem (27.10%)
- MI_Zn_: C ecosystem (30.72%) > B ecosystem (25.04%) > A ecosystem (22.19%)

The lowest values of the mobility index (percentage of bioavailable forms of copper and lead in their total content in soil) in B ecosystem (rural soils) indicate their lowest mobility and bioavailability in comparison to A ecosystem (urban soils) or C ecosystem (roadside soils). Generally, in unpolluted soils, metals occur in stable forms, strongly associated with soil components, and the dominant fraction is the most stable residual fraction. Generally, in unpolluted soils, metals occur in stable forms, strongly associated with soil components, and the dominant fraction is the most stable residual fraction. This is confirmed by other researchers in their publications (Kashem et al., 2007; Lei et al., 2010) that the share of mobile forms in unpolluted soils is usually lower than in contaminated soils with high accumulation of metals. The dominant part of copper in the soils of the Guangdong province in China was in the residual fraction, while its smaller share was recorded in the form associated with Fe–Mn oxides. As much as 86.54% of the total content of lead were forms in the reduction and residual fractions (Xu et al., 2009). Rieuwerts et al. (2006) found that zinc in a labile and bioavailable form constitutes 22–54% of the total zinc content in the soils studied by them. According to them, the dominant forms related to the residual fraction concern not only Zn but also Cu and Pb. The dissolved and exchangeable forms of Cu, Pb, and Zn occur at the lowest concentrations, which is confirmed by the studies of Lei et al. (2010).

### The correlations between the total and EDTA-extractable form concentrations of metals in soils

**Fig. 3:**
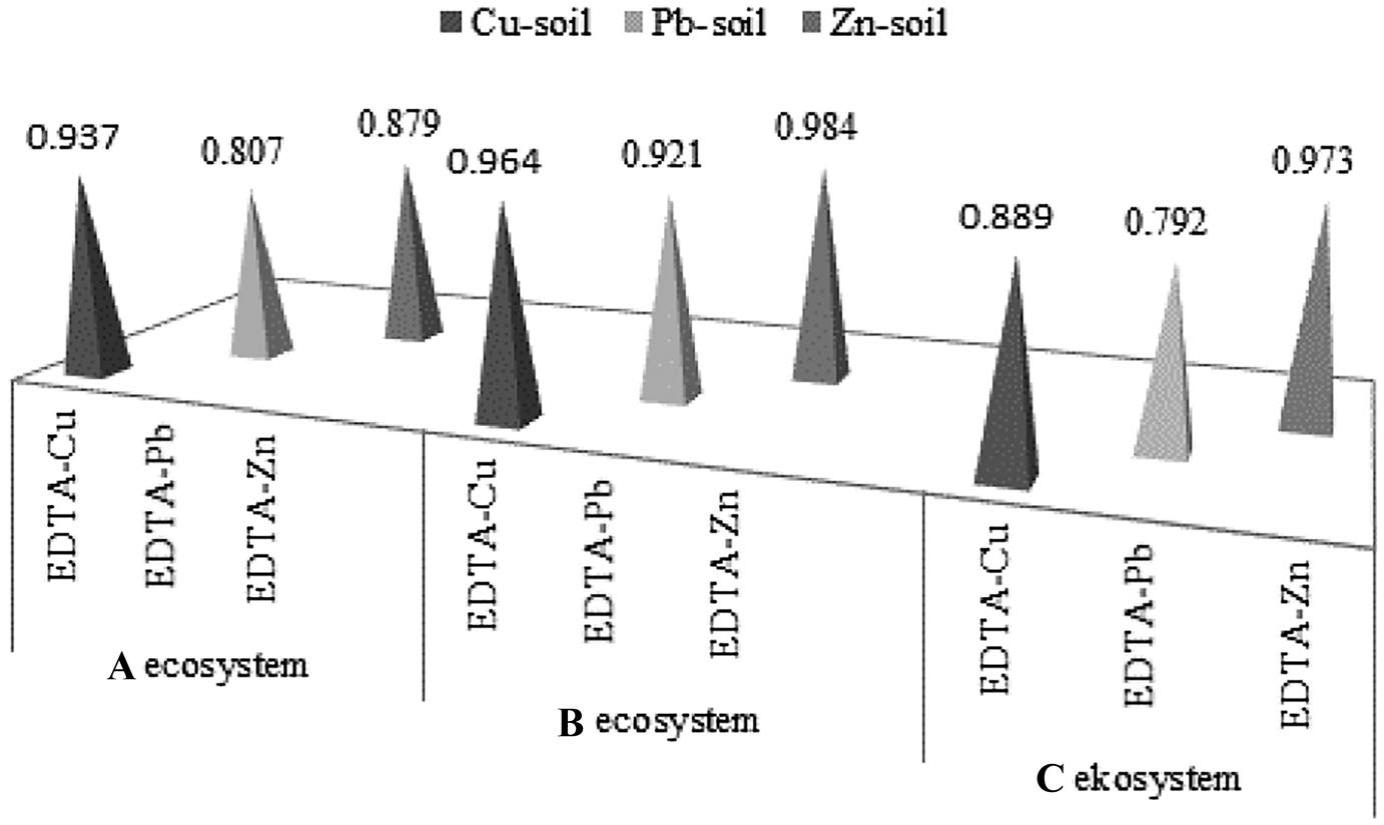
The values of correlation coefficient between the EDTA-extractable form concentrations and the total concentrations of metals in soils

Of the three groups of ecosystems studied (A, B and C), soil samples taken from rural areas (with natural levels of metal accumulation) had the highest values of correlation coefficients (Cu-0.964, Pb-0.922, Zn-0.984) between EDTA-extracted metal concentrations and their total concentrations in soils (Fig. 3). In urban and roadside soils that are subject to anthropopressure, the correlation coefficients between analogous variables were slightly lower (for A/B ecosystems: Cu, 0.937/0.889; Pb, 0.807/0.792; and Zn, 0.887/0.973, respectively). Lower values of correlation coefficients concerned mainly lead and zinc in urban soils and lead and copper in roadside soils. The obtained results may suggest that the continuous inflow of metals from anthropogenic sources to urban and roadside soils may disturb the balance between changeable and non-interchangeable forms of metals in soils exposed to anthropopressure. Similar values of correlation coefficients for lead and zinc were obtained by Anju and Banerjee (2011), which were 0.971 and 0.795, respectively. Positive correlation (statistically significant, many authors emphasize that the concentrations of bioavailable forms depend primarily on the pH of the soil, the content of organic matter or the sorption capacity of the soil (McBride et al., 1997; Kabata-Pendias & Pendias, 2001; Van Gestel, 2008). High values of correlation coefficients between potentially bioavailable species concentrations (as determined by EDTA extraction) and total metal concentrations seem to justify the use of total metal concentrations as a useful preliminary indicator of contamination in areas where the risk of metal excess is high bioavailable with their concentrations in soils (obtained for rural soils—B ecosystem), where the accumulation of metals occurred at the natural level, without a significant share of anthropogenic metals. High values of the correlation coefficients between potentially bioavailable concentrations of species (as determined by EDTA extraction) and total metal concentrations seem to justify the use of total metal concentrations as a useful initial indicator of contamination in areas where there is a risk of excess metals in the soil or, for example, in B ecosystem soils (rural soils), where the accumulation of metals has occurred at natural level, with no significant anthropogenic metal contribution.

### Result of the NIR analysis

The results of the NIR test are presented in Table 3, for which variables and differences between the groups of A, B, and C ecosystems (in three configurations: AB, BC, and AC) are statistically significant taking into account the *p* value.

**Table 3:** LSD test results for selected variables: the total concentrations of metals in soils (Me-soil), the concentrations of EDTA-extractable forms of metals (Me-EDTA), and the values of mobility indexes of metals (MI_Me_)

Variables (df = 51)	Comparison of results (A-B)	Comparison of results (B-C)	Comparison of results (A-C)
Cu-soil	–	–	–
Pb-soil	++++	++++	++
Zn-soil	++++	++	++++
Cu-EDTA	++	++	–
Pb-EDTA	++++	++++	++++
Zn-EDTA	++++	+++	–
MI_Cu_	++	++	–
MI_Pb_	–	–	–
MI_Zn_	++	++	++

Signs: ++++, relevant at level *p* < 0.001; ++, relevant at level *p* < 0.05; +++, irrelevant at level *p* < 0.01; –, irrelevant at level *p* < 0.05

The test shows (Table 3) that the total concentrations of Pb and Zn in the topsoil differed significantly between the groups of ecosystems (A-B, B-C, and A-C), while the differences between the concentrations of Cu were statistically insignificant. Significant differences were found between the concentrations of the bioavailable forms of Cu and Zn in relation to the configuration of the groups (A-B and B-C), while for Pb, the differentiation was significant for each configuration of the groups at the level of *p* < 0.001. The differences between the mobility indices (IM) were statistically significant for Cu in the A-B and B-C configuration (*p* < 0.05) and for Zn also between the A-C groups. There were no significant differences for IM between groups in the case of Pb. The concentrations of Cu, Pb, and Zn forms extracted with EDTA differed significantly between A-B and B-C groups, but no statistically significant differences were found between A-C groups, except for Pb, for which the differences between A-C groups were also statistically significant. The soils of these groups are subject to anthropogenic pressure, unlike the soils representing the ecologically clean B ecosystem. The anthropogenic origin of Cu and Zn in the soils of A and C ecosystems may be the result of the impact of sources of the same nature (e.g. communication), which undoubtedly affect the forms of the deposited metals and how they bind in soil. Therefore, the mean values of the concentrations of the bioavailable forms of Cu and Zn in A and C ecosystems remain at similar levels and do not show significant differences from a statistical point of view. Mobility indices (MI) differed significantly between A-B and B-C ecosystems, while between A-C ecosystems—as in the case of the concentrations of bioavailable forms—no statistically significant differences were found between their mean values. The test results showed no significant differences in the case of Pb in any configuration of group comparisons. The Pb mobility index was not a variable significantly differentiating the affiliation of soil samples to particular of group of ecosystems. In the quantitative category, the most significantly differentiating variables concerned the comparison of the variables of the B ecosystem with the C ecosystem. A similar result was obtained from the comparison of the B ecosystem with the C ecosystem. The smallest differentiating variables were found when comparing soils from A ecosystem with soils from C ecosystem between the groups. This may indicate a similar nature of pollution sources and the degree of influence of metal emitters in these research areas (classified as A and C ecosystems). Statistical analysis (LSD) confirmed the existence of statistically significant differences between the group of unpolluted rural soils of B ecosystem and the groups of polluted soils (A and C ecosystems) under the influence of anthropogenic pressure.

## Conclusion

1. Cu, Pb, and Zn concentrations in the upper soil of the soil representing 2 groups of ecosystems (A and C) significantly exceeded the values of the geological background (Regulation of Minister of Environment, 2016), confirming unequivocally the inflow of metals from anthropogenic sources, typical for areas with a high degree of industrialization (urban soils) and sources related to automotive traffic (roadside soils).
2. The strongest correlations between total metal concentrations in soils and concentrations of EDTA-extracted forms occurred in soils representing B ecosystem (rural soils with natural levels of accumulation of studied metals). The weakening of the correlation strength between the concentrations of bioavailable forms of metals and their total concentrations in soils of the A and C ecosystems (urban and roadside soils) may be the effect of an imbalance between the forms of metal occurrence due to the uncontrolled inflow of metals from anthropogenic sources.
3. Cu and Zn mobility indexes in the studied soils differed significantly between A-B and B-C ecosystems, while between A-C ecosystems—similarly as in the case of bioavailable forms—no statistically significant differences were found. Studies have shown that metals derived from anthropogenic sources are characterized by greater mobility compared to metals of natural origin (lithological associated with the ground). The high similarity between the A and C ecosystems may indicate a similar degree of impact of pollution emitters and the similar nature of pollution sources in areas classified as A and C ecosystems.
4. The inclusion of metal mobility indices in the soil environment in the environmental monitoring strategy may minimize errors in assessing the actual risk associated with the potential uptake of these metals by plants and incorporation into circulation. It may be helpful in estimating and forecasting the risk associated with the transport of trace elements to plants and their toxicity also in long-term conditions.

## Data Availability

The author declares that all other data confirming the results of this study are available in the article.
